# Comprehension of prescription orders with and without pictograms: tool validation and comparative assessment among a sample of participants from a developing country

**DOI:** 10.1186/s12889-023-16856-5

**Published:** 2023-10-05

**Authors:** Nisreen Mourad, Samar Younes, Lidia Mourad, Iqbal Fahs, Shatha Mayta, Racha Baalbaki, Wassim El Basset, Mariam Dabbous, Marwan El Akel, Jihan Safwan, Faraj Saade, Mohamad Rahal, Fouad Sakr

**Affiliations:** 1 School of Pharmacy, Lebanese International University, Bekaa, Lebanon; 2 INSPECT-LB: Institut National de Santé Publique, Épidémiologie Clinique et Toxicologie-Liban, Beirut, Lebanon; 3 School of Pharmacy, Lebanese International University, Beirut, Lebanon; 4 Université de Bourgogne Franche-Comté, PEPITE EA4267, Besançon, France; 5 School of Education, Lebanese International University, Beirut, Lebanon; 6 International Pharmaceutical Federation, The Hague, The Netherlands; 7 Alice Ramez Chagoury School of Nursing, Lebanese American University, Byblos, Lebanon; 8 École Doctorale Sciences de la Vie et de la Santé, Université Paris-Est Créteil, Créteil, France; 9 Institut Mondor de Recherche Biomédicale, UMR U955 INSERM, Université Paris-Est Créteil, Créteil, France

**Keywords:** Pictograms, Medication safety, Medication errors, Comprehension, Medication orders, Health literacy, Patient education, Healthcare

## Abstract

**Background:**

Medication errors can often occur due to the patient’s inability to comprehend written or verbal medication orders. This study aimed to develop pictograms of selected medication orders and to validate the comprehension of prescription orders index and compare the comprehension scores with and without pictograms. In addition to determine the predictors that could be associated with a better or worse comprehension of prescription orders with pictograms versus that of their written counterparts.

**Methods:**

A cross-sectional study was conducted using a snowball sampling technique. Six pictograms were developed to depict specific medication orders. The comprehension of prescription orders index was constructed and validated. The study then compared the comprehension scores of prescription orders with and without pictograms, and identified the predicting factors score difference.

**Results:**

A total of 1848 participants were included in the study. The structure of the comprehension of prescription orders index was validated over a solution of four factors, with an adequate Kaiser-Meyer-Olkin (KMO) measure of sampling adequacy of 0.711 and a significant Bartlett’s test of sphericity (P < 0.001). The construct validity of the index was further confirmed by highly significant correlations between each item and the full index (P < 0.001). The study also found a significant association between the difference in comprehension scores for prescription orders with and without pictograms and several factors, including age, level of education, area of residence, number of children, and smoking status with the difference of comprehension scores (P < 0.001).

**Conclusion:**

Pictogram-based instructions of medication orders were better understood by the Lebanese population than written instructions, making the incorporation of pictograms in pharmacy practice paramount to optimize medication use by the patient and thus yielding better health outcomes.

## Introduction

According to the National Coordinating Council for Medication Error Reporting and Prevention, a medication error is defined as “Any preventable event that may cause or lead to inappropriate medication use or patient harm while the medication is in the control of the healthcare professional, patient, or consumer” [1]. The Institute of Medicine estimated in their report “Preventing Medication Errors” that at least 1.5 million preventable adverse drug events happen each year in the United States alone, making medication errors a major concern in the healthcare sector [2]. In general, medication errors arise during patient care and can occur at any point in the medication process whether in prescribing, transcribing, dispensing, or administration [3]. However, they often occur due to the patient’s inability to comprehend written or verbal medication orders especially among those with low health literacy levels. The inability of patients to understand complicated prescription instructions has actually been identified as an important safety concern and was implicated as an important contributor of medication errors, poor adherence, and detrimental health outcomes like preventable adverse drug reactions, an increase in hospitalizations, and substantial healthcare costs [2, 4, 5]. While having difficulties reading and comprehending prescription instructions has been linked to poor health literacy, it has also been linked to incorrect medication use, poor medication and treatment adherence, poor health outcomes, low quality of life, greater healthcare expenses, and higher rates of all-cause death [6, 7]. Therefore, strategies to reduce medication errors can lead to significant reduction in all of their serious consequences [3].

To address the issue of patients’ comprehension with their prescribed pharmacotherapy, integrating visual tools such as pictograms have been proposed as a viable solution [8, 9]. Pictograms, which come from the Latin word *pictus* meaning “painted” and the suffix–graph meaning “something written,” are graphic representations of concepts or ideas that can be used to communicate messages and information in clear, simple, concise, and expeditious manner to a wide audience irrespective of language, age, or literacy skills [3, 6, 9–14]. In fact, pharmaceutical pictograms have been implemented to help convey medication instructions to patients such as correct use (dose, frequency, administration, duration of treatment,….), and storage of medicine [15, 16]. Pictograms have improved patients’ understanding, retention, recall, and adherence to prescriptions in the medical field [3, 6, 14]. Hence, enabling a decrease in medication errors, an increase in medication accuracy, and an improvement in patients’ disease self-management, all of which result in improved health outcomes [6, 11]. For this reason, both the United States Pharmacopeia (USP) and the International Pharmaceutical Federation (FIP) support the use of pictograms as an effective means of communication to convey crucial information on medication utilization [11, 17].

A pictogram should be viewed as a two-part construct consisting of a symbol (the visual representation) and a referent (the intended meaning). It is important to note that the referent can have different interpretations depending on context and culture, so both must be taken into account when designing and employing a pictogram [3, 6, 18]. Since certain symbols or colors may have different meanings across cultures, there has been evidence that pictogram comprehension varies significantly between cultures and countries. This variation in pictorial interpretation can lead to misunderstandings and confusion [6, 11, 19]. Thus, the development and implementation of pictogram-based instructions require consideration of the target population’s culture, beliefs, and attitudes [7].

The development and testing of pharmaceutical pictograms involve a stepwise approach and a rigorous process to ensure that the symbols are clear, understandable, and culturally appropriate [3, 14, 18]. The initial phase of development involves identifying the needed medication instructions to be communicated with the target population, then creating an initial set of pictograms based on established principles of design and communication, followed by testing and validating them for comprehension and appropriateness to convey the intended message within the target audience [14]. The FIP states that transparency and translucency are two distinct attributes that can be used to assess pictogram comprehension [7]. Transparency is the ability to deduce the meaning of a picture or image while translucency is the extent to which a participant, after being informed of the meaning of the pictogram, feels that the image depicts what it is intended to represent [7]. It is important to note that research has shown that pictograms with written text are preferred since they offer optimal processing and enhance recall of medication information [15, 20].

There is currently no published research on pictograms in Lebanon or their use for rendering medication instructions easier to understand. As such, the aims of the current study were to develop pictograms of selected medication orders and to differentiate their comprehensibility versus that of conventional text among the Lebanese population. Moreover, the study intended to explore the demographic and socioeconomic factors that affected the pictogram comprehension among the intended population. The findings of the study will have implications on both patients and healthcare providers, with the potential to encourage the use of culturally appropriate pictogram-based instructions as an effective means of communicating medication information and improving patient outcomes.

## Methods

### Study design and variables

**Fig. 1:**
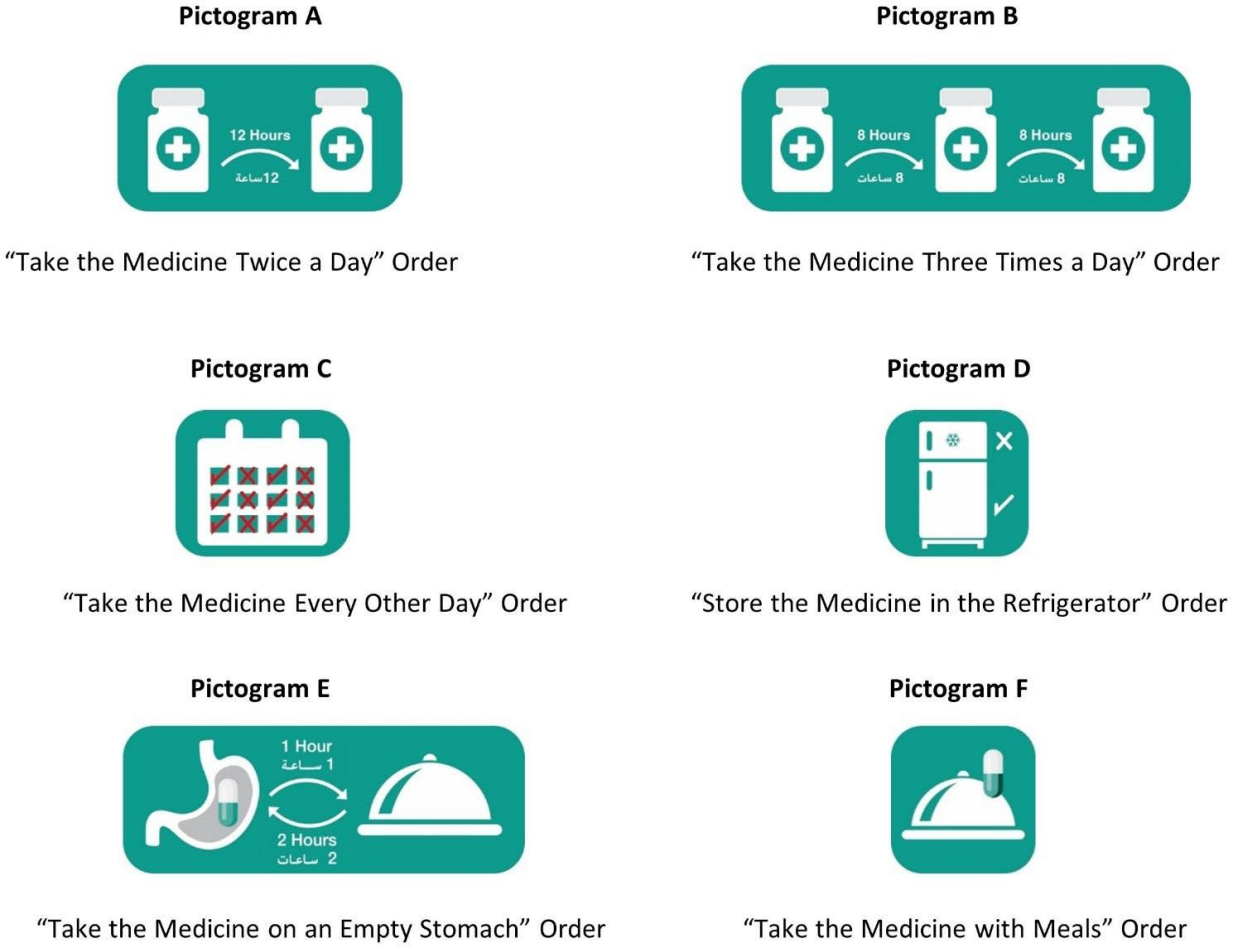
Pictograms illustrating selected medication orders

A cross-sectional study was conducted using a snowball sampling technique between August 2022 and January 2023. Six pictograms illustrating selected medication orders were developed by the research team (Fig. 1). Thereafter, an anonymous, self-administered, web-based questionnaire was disseminated in which the study’s aims were explained. The questionnaire was divided into 2 sections. The first section included questions about the sociodemographic characteristics of participants such as age, gender, area of residence, level of education, marital status, number of children, social history, and presence of chronic diseases. The second section included 12 questions to composite the “comprehension of prescription orders index” based on a comprehensive literature review. The first part of the index comprised the assessment of comprehension of prescription orders without pictograms subscale. This subscale included 6 positively phrased questions to assess prescription orders comprehension without pictograms using multiple choice options with one correct answer. Questions included the meaning of taking a medicine two times per day, three times per day, every other day, on an empty stomach, or with meals, in addition to the meaning of storing a medicine in the refrigerator. The second part of the index comprised the assessment of comprehension of prescription orders with the pictograms’ subscale. This subscale required the participants to define the instructions of the 6 developed and described pictograms (Fig. 1), which also used multiple choice questions with a single right response.

### Outcome measures

The primary outcome measure was to validate the comprehension of prescription orders index and compare the comprehension scores with and without pictograms with the aim to understand the role of pictograms in the process of patient care. The secondary outcome measure was to determine the predictors that could be associated with a better or worse comprehension of prescription orders with pictograms versus that of their written counterparts.

### Sample size calculation

The minimal required sample size was calculated using CDC Epi-Info for population surveys. The expected frequency was set at 50% to yield the largest possible minimal sample size. Accordingly, a minimum sample of 384 participants was required to allow for adequate power of statistical analysis, and produce a 95% confidence level and an acceptable margin of error of 5%. For the validation of the comprehension of prescription orders index, a ratio of participants to items should be at least 10:1 according to the rule of thumb [21]. The rationale behind this rule is to ensure that the sample size is a sufficiently large sample to obtain stable parameter estimates. When the sample size is relatively smaller to the number of parameters, the model may become overfit, leading to poor generalizability of the results. Therefore, the rule of thumb with a 10:1 ratio ensure that the model is more likely reflecting the true population relationships between variables [22]. Thus, a minimal sample size of 120 participants was required to validate the comprehension index. A larger sample size was targeted to allow for additional analyses.

### Ethical aspects

This study was performed in line with the principles of the Declaration of Helsinki and was approved by the Ethics and Research Committee of the School of Pharmacy at the Lebanese International University (Protocol number: 2023RC-015-LIUSOP). Participants’ privacy, anonymity, and confidentiality were warranted as personal identifiers of participants were not traced. All participants who agreed to participate in the study provided informed consent.

### Statistical analysis

The data were analyzed using SPSS version 26.0. The analysis involved presenting descriptive statistics in terms of frequencies and percentages for categorical variables, and means (± standard deviation, SD) for continuous variables. To validate the structure of the comprehension of prescription orders index, factor analysis was conducted using principal component analysis (PCA) with Promax rotated component matrix. The model adequacy was confirmed by measuring the Kaiser-Meyer-Olkin (KMO) measure of sampling adequacy, and the Bartlett’s test for sphericity. The internal consistency and reproducibility of the index was confirmed by measuring Pearson correlation coefficients between each item of the index with its corresponding subscale, as well as with the overall index. The comprehension scores of prescription orders with and without pictograms were compared using paired sample T-test. The difference in the comprehension scores with and without pictograms was computed. To determine the predictors of the score difference, a multivariable linear regression analysis using a backward stepwise approach was performed taking the computed comprehension score difference as the dependent variable, and the sociodemographic characteristics of participants as independent variables. Results were reported as unadjusted beta and 95% confidence interval. The level of significance was set at P < 0.05 with a margin of error equal to 5%.

## Results

### Sociodemographic characteristics

A total sample of 1848 participants were reached. The mean age of the participants was 29.52 ± 12.68 years, 64.7% were females, 91.5% were Lebanese, and 21.1% were residing in the Bekaa region. For the level of education, 73.4% had a university degree, and greater than half of the participants were single and had no children. Around 60% of the participants never smoked and the majority were non-alcoholic and had no chronic diseases (88.3% and 87.2% respectively). Table 1 shows the detailed sociodemographic characteristics of the participants.

**Table 1:** Sociodemographic characteristics of the participants

Variable	N (%)
**Gender**	
Female	1196 (64.7%)
Male	652 (35.3%)
**Nationality**	
Lebanese	1689 (91.4%)
Non-Lebanese	159 (8.6%)
**Area of residence**	
Akkar	76 (4.1%)
Baalbek-Hermel	99 (5.4%)
Beirut	280 (15.2%)
Bekaa	390 (21.1%)
Mount Lebanon	326 (17.6%)
Nabatieh	228 (12.3%)
North	225 (12.2%)
South	224 (12.1%)
**Level of education**	
Primary school	116 (6.3%)
High school	317 (17.2%)
University	1356 (73.4%)
No education	59 (3.2%)
**Marital status**	
Single	1119 (60.6%)
Married	679 (36.7%)
Separated/Divorced/Widowed	50 (2.7%)
**Number of children**	
0	1118 (60.5%)
1 to 2	362 (19.6%)
3 and more	368 (19.9%)
**Smoking status**	
Current	469 (25.4%)
Never	1132 (61.3%)
Former	247 (13.4%)
**Alcohol use**	
Yes	216 (11.7%)
No	1632 (88.3%)
**Employment status**	
Full-time	335 (18.1%)
Part-time	574 (31.1%)
Unemployed	939 (50.8%)
**Household monthly income**	
< 3,000,000 LBP	216 (11.7%)
3,000,000–5,999,999 LBP	283 (15.3%)
6,000,000–8,999,999 LBP	272 (14.7%)
9,000,000–14,999,999 LBP	265 (14.3%)
15,000,000–29,999,999 LBP	145 (7.8%)
≥ 30,000,000 LBP	83 (4.5%)
Don’t know/No response	584 (31.6%)
**Presence of chronic diseases**	
Yes	236 (12.8%)
No	1612 (87.2%)
	**Mean ± SD**
**Age**	29.52 **± 12.68**

### Validation of the comprehension of prescription orders index

#### Factor analysis

Factor analysis was run to validate the structure of the comprehension of prescription orders index. All items could be extracted with Promax rotation with an adequate Kaiser-Meyer-Olkin (KMO) measure of sampling adequacy equal to 0.711 and a significant Bartlett’s test of sphericity (P < 0.001). The items loaded on 4 factors with Eigenvalue greater than 1 and explaining 58.51% of the total variance. Table 2 shows the Promax rotated matrix of the comprehension of prescription orders index.

**Table 2:** Promax rotated matrix of the comprehension of prescription orders index

	Factor 1 loading	Factor 2 loading	Factor 3 loading	Factor 4 loading
Pictogram A meaning	0.790			
Pictogram B meaning	0.713			
Pictogram E meaning	0.703			
Pictogram C meaning	0.668			
Taking a medicine three times per day meaning		0.829		
Taking a medicine two times per day meaning		0.821		
Taking the medicine every other day meaning		0.648		
Taking a medicine on an empty stomach meaning		0.552		
Taking a medicine with meals meaning			0.814	
Pictogram F meaning			0.808	
Storing a medicine in the refrigerator meaning				0.868
Pictogram D meaning				0.667

Kaiser-Meyer-Olkin (KMO) measure of sampling adequacy = 0.711.

Bartlett’s test of sphericity P < 0.001.

Percentage of total variance explained = 58.51%.

#### Validity measures

The construct validity of the comprehension of prescriptions index was further confirmed by measuring the correlation of each comprehension assessment item with its subscale and with the total comprehension index. All items significantly correlated with their subscales and with the total index. The Pearson correlation coefficient ranged from 0.241 to 0.750 for the comprehension of prescriptions without pictograms items with their subscale, 0.391 to 0.704 for the comprehension of prescriptions with pictograms items with their subscale, and 0.283 to 0.673 for all items with the total index. The Pearson correlation coefficient of the comprehension of prescription without and with pictograms subscales with the total index were 0.908 and 0.662 respectively. Table 3 presents the Pearson correlation of the comprehension of prescriptions index.

**Table 3:** Pearson correlation of the comprehension of prescription orders index

Scale Item	r 1^*^	r 2^*^	r 3^*^
Taking a medicine two times per day meaning	0.672^**^	0.750^**^	
Taking a medicine three times per day meaning	0.642^**^	0.742^**^	
Taking the medicine every other day meaning	0.554^**^	0.627^**^	
Storing a medicine in the refrigerator meaning	0.398^**^	0.406^**^	
Taking a medicine on an empty stomach meaning	0.463^**^	0.547^**^	
Taking a medicine with meals meaning	0.286^**^	0.241^**^	
Comprehension of prescriptions without pictograms subscale	0.908^**^		0.288^**^
Pictogram A meaning	0.483^**^		0.704^**^
Pictogram B meaning	0.393^**^		0.577^**^
Pictogram C meaning	0.409^**^		0.623^**^
Pictogram D meaning	0.311^**^		0.523^**^
Pictogram E meaning	0.373^**^		0.606^**^
Pictogram F meaning	0.283^**^		0.391^**^
Comprehension of prescriptions with pictograms subscale	0.662^**^	0.288^**^	

^*^r 1 = Pearson correlation coefficient with the comprehension of prescriptions full scale; r 2 = Pearson correlation coefficient with the comprehension of prescriptions without pictograms subscale; r 3 = Pearson correlation coefficient with the comprehension of prescription with pictograms subscale.

^**^P < 0.001.

### Comparison of comprehension scores with and without pictograms

**Fig. 2:**
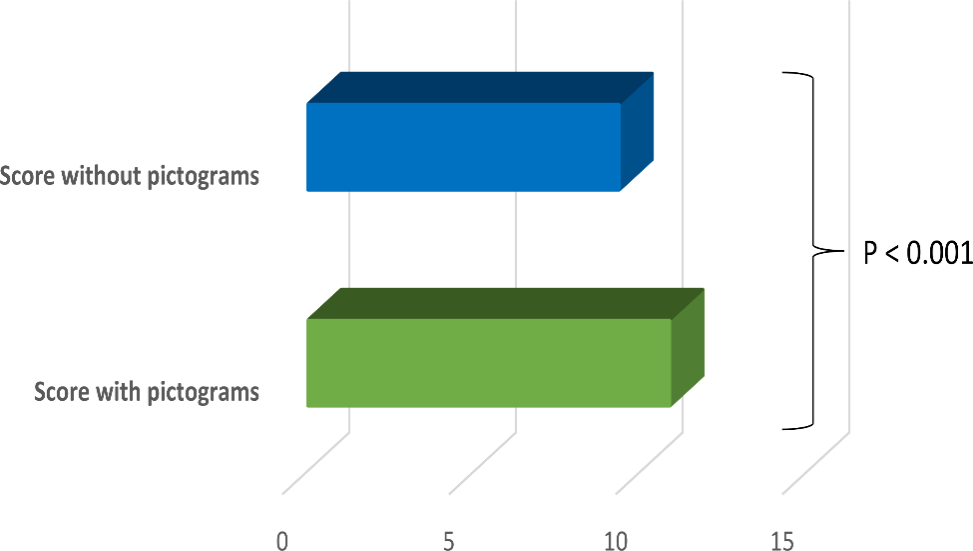
Comparison of prescription orders comprehension scores with and without pictograms

The comprehension of prescription orders with and without pictograms scores were computed by summing up the correct answers of items in each subscale. The comprehension score of prescription orders was significantly higher with pictograms compared to that without pictograms with a mean difference of 1.52 (95% CI 1.45; 1.60, P < 0.001). The mean scores of the prescription orders comprehension with and without pictograms were 10.88 (± 0.89) and 9.36 (± 1.60) respectively (Fig. 2).

### Predictors of comprehension score difference with and without pictograms

A multivariable linear regression was performed to determine the predictors of score difference with and without pictograms. A backward linear regression model was run taking the difference between the comprehension scores with and without pictograms as the dependent variable, and the sociodemographic characteristics of participants as independent variables. There was a significant negative association between age and the difference of comprehension scores. Older participants had a significantly lower score difference compared to younger participants (Beta = -0.019, P < 0.001). On the other hand, there was a significant positive association between the level of education, area of residence, number of children, and smoking status with the difference of comprehension scores. Participants with primary school education (Beta = 0.675, P < 0.001) and high school education (Beta = 0.430, P < 0.001) had significantly higher score difference compared to non-educated participants. Former smokers had significantly higher score difference compared to current smokers (Beta = 0.365, P < 0.001). Participants with 1 to 2 children had also significantly higher score difference compared to participants with 3 or more children (Beta = 0.239, P = 0.046). Finally, participants residing in Mount Lebanon (Beta = 0.550), Nabatieh (Beta = 0.787), and North of Lebanon (Beta = 0.682) had significantly higher score difference compared to participants from Akkar (all P values < 0.001). The multivariable linear regression analysis taking the difference between the prescription orders comprehension scores with and without pictograms as the dependent variable is shown in Table 4.

**Table 4:** Multivariable linear regression analysis taking the difference between the prescription orders comprehension scores with and without pictograms as the dependent variable

Variable	Unstandardized Beta	Standardized Beta	P value	95% CI	
				Lower Bound	Upper Bound
Age	-0.019	-0.148	< 0.001	-0.028	-0.009
Gender (male vs. female)	0.144	0.043	0.053	-0.002	0.290
Area of residence (reference: Akkar)					
Mount Lebanon	0.550	0.132	< 0.001	0.361	0.739
Nabatieh	0.787	0.162	< 0.001	0.563	1.010
North	0.682	0.140	< 0.001	0.459	0.905
Level of education (reference: No education)					
Primary school	0.675	0.103	< 0.001	0.378	0.973
High school	0.430	0.102	< 0.001	0.245	0.615
Marital status (reference: Single)					
Married	0.266	0.081	0.066	-0.017	0.550
Separated/Divorced/ Widowed	0.479	0.049	0.066	-0.031	0.989
Number of children (reference: ≥ 3)					
0	-0.290	-0.089	0.076	-0.610	0.030
1 to 2	0.239	0.059	0.046	0.004	0.474
Smoking status (former vs. current smoker)	0.365	0.078	0.001	0.156	0.574

## Discussion

Medication error management has undergone a positive evolution toward a systematic approach that identifies and addresses the underlying causes [23]. This has led to the provision of tools and resources to help reduce medication administration errors [24]. Pictograms can serve as an interesting method to decrease such errors and their subsequent consequences because of their visual effect and their ability to convey meaningful information in a precise and concise way [25]. This study is one of the first studies in Lebanon that sheds light on the importance of pictograms. Pictograms of selected medication orders were designed and their comprehensibility versus that of conventional text among the Lebanese population was compared. The results revealed a significantly higher comprehension score of prescription orders with pictograms.

The comprehensibility of the prescription orders with or without pictograms in this study was assessed using a developed “comprehension of prescription orders index”. This index was validated for structure using factor analysis, with an adequate KMO measure of sampling adequacy and a significant Bartlett’s test of sphericity. The items loaded on 4 factors and Eigenvalue greater than 1 thus explaining 58.51% of the total variance. In addition, all items were significantly correlated with their subscales and with the total index. Though being a difficult and time-consuming process, designing, testing and validating pictograms is extremely essential to ensure their suitability, culture-sensitivity, and to evaluate how our patient population comprehends them. The current study applied a rigorous approach to validate the pictograms instrument by applying factor analysis to validate the structure of the instrument and examining the correlation of each item with the whole pictogram instrument. However, in the absence of any published gold standard on pictogram instruments validation, this study was not able to go for further validity measures including the computation of sensitivity and specificity of the current instrument. Further research is suggested in this context to provide additional validity measures on such instruments.

The comprehension score of prescription orders in our study was significantly higher with pictograms compared to written orders without pictograms. Written health information, including prescription orders, offers challenges and barriers even to literate readers [26]. On the other hand, pictograms with their visual content enhance the reader-friendliness of the information as well as improve the comprehension and recall of medicines and general health information [26]. Moreover, pharmaceutical pictograms were more valuable when used in combination with verbal and written reinforcement [27]. In a study where participants were randomly allocated to a control text-only information or experimental group with text and pictogram information, both sets were generally well understood [28]. However, the presence of pictograms had a positive effect on the acquisition and comprehension of drug information [28], which are in accordance with our results. Though, in our study, the same group of patients were asked to comprehend a written prescription order and then a pictogram for the written order. This helps to alleviate the impact of various confounding variables of two different groups on the comprehensibility scores. In another study, when selected pharmaceutical pictograms were evaluated, the majority of the patients were unable to interpret the meaning of pictograms correctly before explaining their meaning [29]. After explanation, interpretation of the meaning of pictograms comparatively improved indicating the need of using pictograms along with verbal reinforcement [29]. This was unlike our study where we compared the comprehension of written orders without pictograms versus with pictograms. No assessment of verbal counseling or reinforcement was done in this study as the data collection was done via web-based platforms and not face-to-face interviews.

Several demographic and socioeconomic factors that affected the comprehension of pictograms among the Lebanese population were identified in this study. A multivariable linear regression demonstrated a significant negative association between age and the difference of comprehension scores, with older participants having significantly lower score difference compared to younger ones. This means that older age was associated with lower comprehension of pictograms. Low health literacy, high medication burden and poly-pharmacy, visual disorders, and cognitive aging in the older adult population are contributing factors to the misunderstanding of medication instructions [30–32]. Similar to our results, low comprehension of pictograms was observed among older Singaporeans with limited English proficiency [33]. Also, a study that assessed the understanding and cultural acceptability of the United States Pharmacopeia Dispensing Information (USP-DI) in a group of elderly Brazilians, found that most of the USP-DI pictograms evaluated were not well understood by the elderly Brazilians [34]. Such results emphasize the importance of facilitating pictogram understanding during medication counseling for older patients. Also, in a descriptive cross-sectional research among adults in the Philippines, the median score of pictograms that passed the American National Standards Institute (ANSI) criterion of 85% comprehension was 1.20 points lower if the patient was greater than 46 years old, but the difference was not statistically significant [35]. This could be attributed to possible visual impairment which was not a factor in that study because the pictograms were printed largely on the flipbook during data collection [35]. Also, contrary to our results, in a study that enrolled older adults aged 65 or older from one community pharmacy in Canada, no association between initial comprehensibility of the pictograms and age was found [36]. This could be due to better visual acuity, health literacy, education, support and counseling provided to those patients in Canada.

On the other hand, there was a significant positive association between the level of education and the difference of comprehension scores. Participants with primary and high school education had significantly higher score differences compared to non-educated participants. Similarly, the result of a study conducted in the Philippines, suggested that higher education translated to a higher comprehension of pictograms [35]. The median score of pictograms that passed the ANSI criterion of 85% comprehension was 21.58 points lower if the participant was below Grade 12 education level, and the education level was identified as a significant predictor [35]. Also, in a previous South African study, significant differences in interpretation of pictograms were apparent with only primary school education and those who had completed at least some senior school education [37]. The group with tertiary education was significantly better than the other groups. Hence, to increase comprehension, training and patient education are necessary to ensure the effectiveness of symbols and pictograms [14]. Therefore, the inclusion of topics related to health pictograms in basic health education in Lebanon is recommended. Alternatively, since verbal instructions were better comprehended by individuals with low literacy skills than pictograms, pictograms must be complemented with verbal and written instructions during patient counseling [38].

Furthermore, former smokers had significantly higher score differences compared to current smokers. This could be due to better health literacy and exposure to healthcare providers and health information among former smokers compared to current smokers. Finally, participants residing in Mount Lebanon, Nabatieh, and North of Lebanon had significantly higher score differences compared to participants from Akkar. This could be explained by the lower socioeconomic status in Akkar district compared to other areas in Lebanon.

### Strengths and Limitations

Several limitations to our study should be highlighted. First, the survey administered was self-reported by the participants. So, respondents might tend to avoid extreme responses or might exhibit acquiescence. Also, this was an observational study; hence, it does not prove cause and effect relationships. Among the limitations of the current study, as well, is the fact that we did not assess participants’ visual acuity. It is possible that some participants did not understand some of the pictograms because of vision problems. In addition, we did not assess health literacy, especially that pictograms are often implemented to help people with low levels of health literacy. Moreover, we were not able to assess the impact of verbal counseling or reinforcement since the data collection was done via web-based platforms and not face-to-face interviews. Also, other factors that might affect comprehension scores were not explored in this research, such as access to health information materials, access to the internet, and participation in health programs among others. Finally, the order of the questions could play a role in the difference in the scores due to a possible risk of information bias. In the first section, patients were asked without pictograms. This could have made their responses better for the second question when they got pictograms. However, it is believed that this risk is minimized as the questions without and with pictograms were on different sections of the questionnaire so that respondents will not have a direct link between each question without and with pictograms.

Despite these limitations, this is the first research paper in Lebanon that assessed the comprehension of pictograms along with the associated factors. The sample size, as well, was relatively huge and thus provided power for adequate statistical analysis. Moreover, the data collection was done from all Lebanese districts which allows generalization of results to the Lebanese population. Also, a new index tool the “comprehension of prescription orders index” was designed and validated to assess the order comprehension and utilized to evaluate the impact of pictograms on comprehension scores. Furthermore, the findings offered recommendations for future researchers to improve the comprehension of pictograms in Lebanon, including:

- Develop and design pictograms adapted to the Lebanese culture while following the best practices in writing health education material.
- Combine the pictograms with written instructions and verbal counseling to ensure adequate comprehension especially when dealing with elderly patients and patient with low educational levels.
- Include pictograms in the Lebanese basic health education to be effectively and safely used in patient information materials.

## Conclusion

In conclusion, pictogram-based instructions of medication orders were better understood by the Lebanese population than their written counterparts. This highlights that pictograms offer a universal language that surpasses linguistic and cultural barriers making their incorporation in pharmacy practice paramount to optimize medication use by the patient and minimize the risks of errors thus yielding better health outcomes.

## Data Availability

The datasets used and/or analysed during the current study are available from the corresponding author on reasonable request.
